# An In Vitro Study Comparing the Diametral Tensile Strength of Composite Core Build-Up Material With Three Different Prefabricated Post Systems

**DOI:** 10.7759/cureus.29560

**Published:** 2022-09-25

**Authors:** Kamal K Meena, Vineet Sharma, Ratnesh K Jaiswal, Rahul Madaan, Madhukar Gupta, Surendra Jaswal

**Affiliations:** 1 Prosthodontics, Rajasthan University of Health Sciences (RUHS) College of Dental Sciences, Jaipur, IND; 2 Periodontics, Rajasthan University of Health Sciences (RUHS) College of Dental Sciences, Jaipur, IND; 3 Dentistry, Community Health Center, Raniwara, Jalore, IND; 4 Oral Medicine and Radiology, Pacific Dental College & Hospital, Udaipur, IND

**Keywords:** glass fiber post, carbon fiber post, stainless steel post, composite core, diametral tensile strength

## Abstract

Statement of problem

Dental restorations are subjected to tensile stresses from oblique or transverse loading of their complex geometric forms, making tensile strength a fundamental mechanical property. Since composite core build-up materials are brittle, the integrity of the post and core depends on their tensile strength and resistance to fracture when utilized with various prefabricated post systems. Therefore, it is essential to determine the tensile strength of the prefabricated metallic and nonmetallic posts used to reinforce the composite resin core.

Purpose

This study compared the diametral tensile strength (DTS) of three prefabricated post systems with composite core build-up material.

Material and methodology

Ten composite resin cores from four different groups were formed. The control group was the composite resin core without a post (group 1). Group 2 was composed of composites with metal posts, group 3 was composed of composites with glass fiber posts, and group 4 was composed of composites with carbon fiber posts. All the samples were kept in a humid place for seven days to mimic the conditions in the mouth. DTS was determined by recording the tensile force required to fracture the core material by performing a diametral compression test for tension after a week. The observations were analyzed using a one-way analysis of variance (ANOVA), followed by a post-hoc test.

Results

The tensile strength of the resin core material was decreased by 28.1%, 20.8%, and 10.4% by using posts made of stainless steel, carbon fiber, and glass fiber, respectively. Among the three post systems, stainless steel had the lowest mean DTS values, while glass fiber had the highest mean DTS values.

Conclusion

Composite core glass fiber post systems showed higher tensile strength than other post systems.

## Introduction

The efficacy of a pulpless tooth to function harmoniously in the oral cavity depends on an effective endodontic treatment followed by an equally effective restorative procedure. The first goal of restoring an endodontically treated tooth is reinforcing the remaining tooth structure, replacing the missing tooth structure, or both. The second goal is to make the design and the final restoration easier, which should protect the tooth and conform around it [1].

The restoration work must use a dowel (post), a core, and a crown [2]. The dowel, also called "post," is usually made of metal and is placed into a natural tooth's root canal after the canal has been prepared [3]. The combination of a post and a core gives the restoration both stability and strength. The goals of designing the post, the core, and the crown are to disperse the stresses caused by torque across the rest of the tooth structure. The prefabricated posts had some advantages, such as being more homogeneous, having standard sizes and shapes, decreasing chair-side time, and being easy to manipulate. Several in vitro and clinical studies have revealed that cast post is ineffective [4]. It could be due to the wedging effect and the increased risk of root fracture. Today, prefabricated posts are available in many materials, including metal, carbon, glass, and ceramic. Fiber posts offer good esthetics and modulus of elasticity values such as dentin, resulting in a more uniform stress distribution on the root and a lower incidence of root fractures [5,6]. Ceramic posts provide high esthetics and strength, but their modulus of elasticity is greater than dentin and predisposes them to root fractures, especially in thin roots [7].

The core components can be cast alloys, amalgams, glass-ionomer cement, or resin composites [8]. Compared to amalgam and glass ionomers, composite resin has become a preferred core material due to its strength, ease of manipulation, and rapid setting time. Teeth can be prepared for crown restoration at the same session due to the quick hardening. Composite resins can bond to posts and crowns when proper bonding processes are applied [9]. A dual-cured composite resin is easy to manipulate, polymerizes quickly, and allows deeper layers that are not visible to the curing light to cure chemically. This dual-cure feature gives it an edge over exclusively light-cured materials, which are limited by cure depth.

Tensile forces are applied to the entire assembly (post and core) after being inserted into the oral cavity. The American Dental Association specifications for direct resin composites specify minimum diametral tensile strength (DTS) values ranging from 24 Megapascal (MPa) to 34 MPa [10]. Still, there is a need to determine the strength of resin composite cores reinforced with currently available prefabricated metallic and nonmetallic posts. This in vitro study evaluated the DTS of three different post systems attached to composite core material. The null hypothesis stated that there was no change in the dimensional tensile strength of the resin composite core material with or without post reinforcement.

## Materials and methods

The diametral compression test is a popular method for assessing brittle material tensile strength because it eliminates some of the issues inherent in direct and flexural tensile testing and due to its relative simplicity and consistency of results.

Custom-made mold

**Figure 1:**
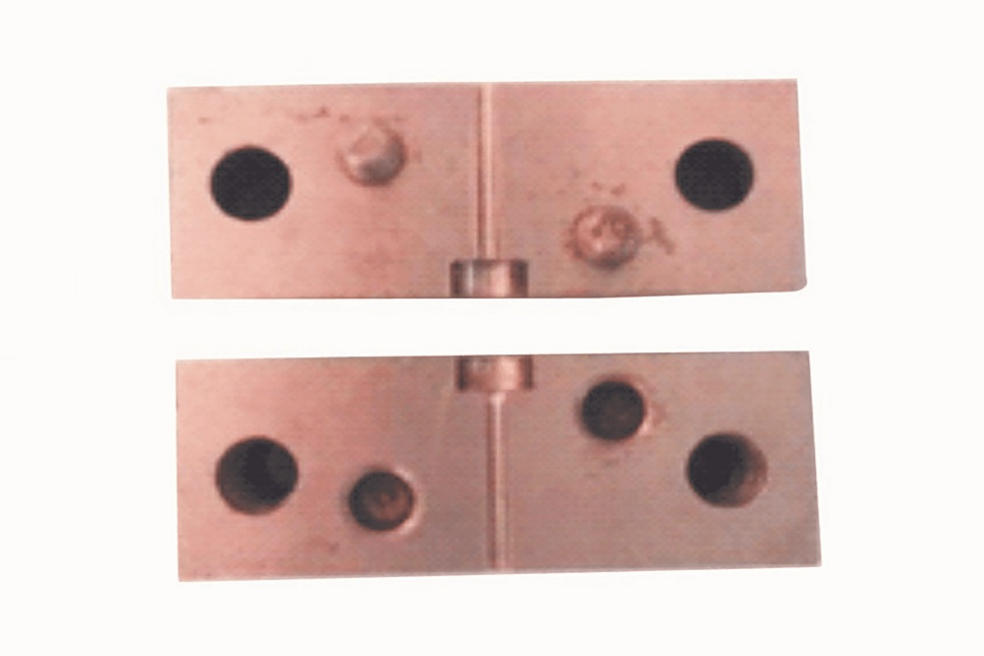
Stainless steel mold

A custom-made stainless steel mold was prepared to make the post/core specimens with two split compartments: a lower compartment (base) with a channel hole 1.5 mm in diameter and 15 mm in height to receive the post and an upper compartment with a perforation 6 mm in diameter and 3 mm in height centered over the perforation (channel hole) in the lower compartment (Figure 1). The two compartments were secured together with screws. As a result, a post could be placed in the mold to fit into a channel hole in the lower compartment and be centered in the upper compartment hole. The cylindrical hole in the upper compartment was then filled with a core.

Core build-up material

This study used a dual-cured, radio-opaque composite resin core build-up material (ParaCore, Coltène/Whaledent Private Ltd., Mumbai, India) supplied in a syringe system. It can also be used to cement root posts and indirect restorations. Chemical-curing adhesives (adhesives A and B) and a non-rinse conditioner were included in the composite kit.

Preparation of test samples

After placing the post in the channel, the manufacturer's instructions for core material regarding conditioning, adhesive application, and curing were followed. The core material was extruded into the cavity in slight excess, and to prevent air inclusion, finger pressure was applied with a Mylar strip film over the cylindrical mold. The samples were subjected to a VLC (visible light cure) polymerization unit with a light intensity of 800 mW/cm^2^ for 40 seconds. The post-core assemblies were removed from the molds by tapping them from the apical end of the post.

**Figure 2:**
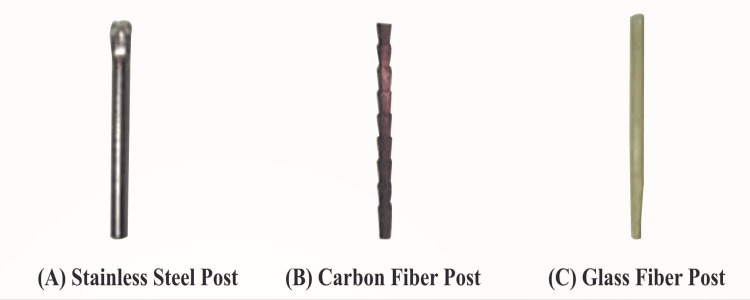
Prefabricated post systems

**Figure 3:**
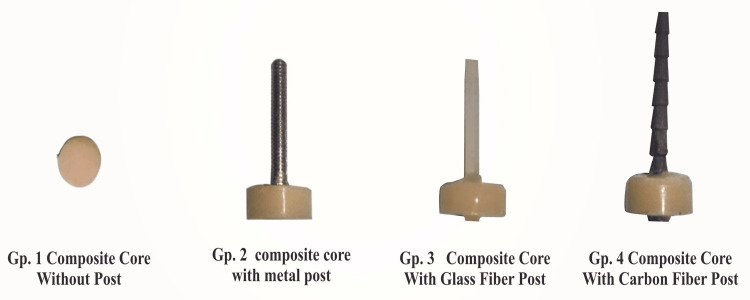
Four groups

**Figure 4:**
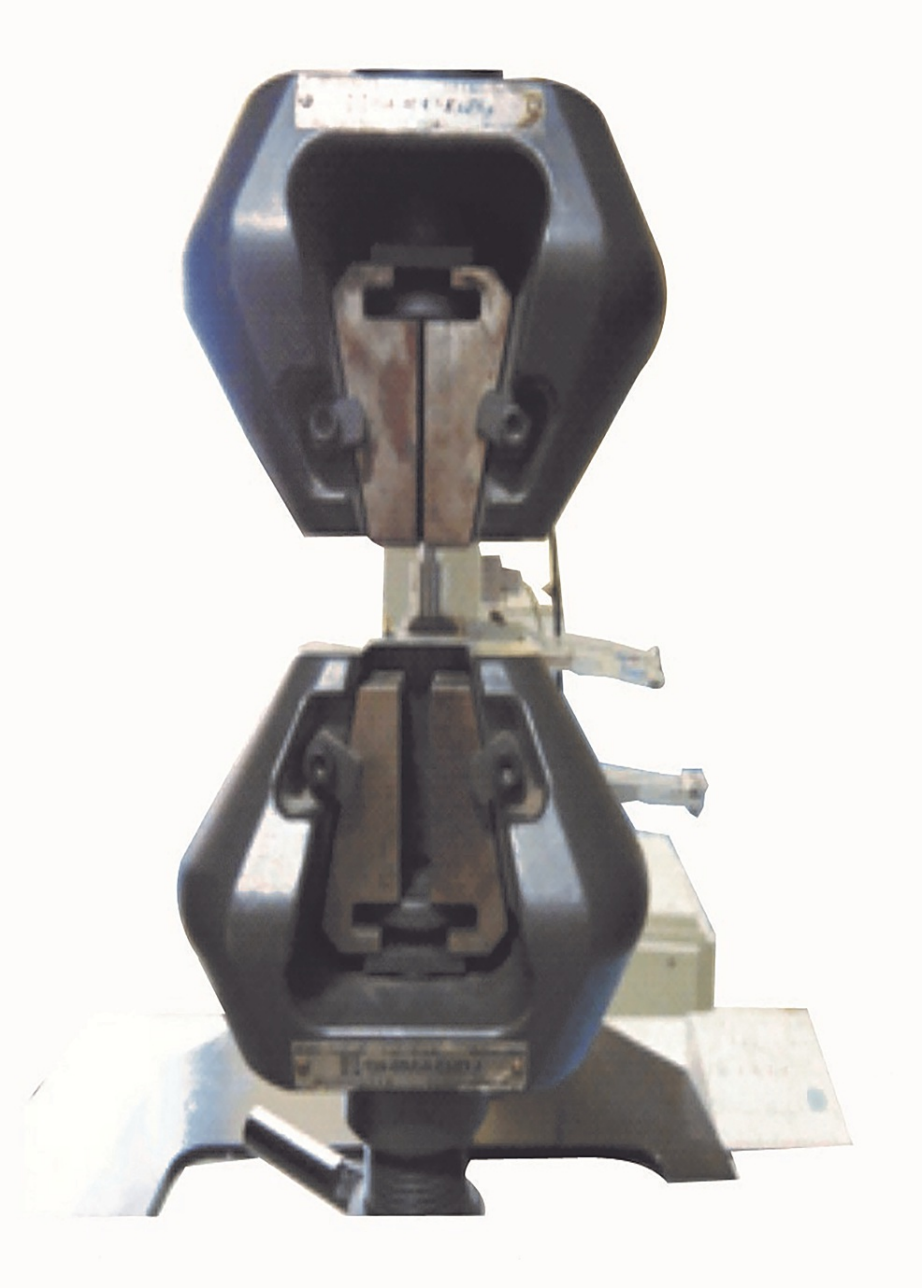
Universal testing machine

The post and core samples were divided into four groups: resin core without a post as the control group (group 1), stainless steel post (group 2) (ParaPost System, Coltène/Whaledent Private Ltd., Mumbai, India), glass fiber post (group 3) (TENAX® Fiber Trans, Coltène/Whaledent Private Ltd., Mumbai, India), and carbon fiber post (group 4) (Reforpost Glass Fiber, Angelus, Londrina, Brazil) (Figures 2, 3). To imitate oral conditions, all samples were stored in a humid environment, i.e., distilled water at 37°C for seven days. The post-core assemblies were taken out for testing after a week. The tensile force necessary to fracture the core material was recorded using a diametral compression test for tension to determine the diametric tensile strength. For this purpose, a universal testing machine (UTM; AG-IS Shimadzu Universal Testing Machines, Shimadzu Analytical India Pvt. Ltd., Mumbai, India) was suitable (Figure 4). The machine delivered force to each specimen in compression mode at a crosshead speed of 1 mm/minute and a load cell of 2,000 kg. Samples were engaged between the UTM's two plates. Each disc-shaped specimen was oriented horizontally on the machine platform so that the loading plate surfaces made tangential contact with specimens. The two plates supplied an equal and opposing compressive force to the post and core assembly, causing stresses within the specimen along a vertical plane until visible or audible evidence of failure was demonstrated. The results were received on the machine's display panel, and the DTS was calculated using software already installed in UTM. The mean values and standard deviations were calculated for each group. The data were analyzed using a one-way analysis of variance (ANOVA) to see if there was a significant difference between the mean values of the four groups, followed by the post-hoc test (p = 0.05) to examine the intragroup comparison between the specific groups.

## Results

Group 1 had the greatest mean DTS value (51.65 MPa) among all groups, while group 2 had the lowest mean DTS value (37.14 MPa). Group 3 has a higher mean fracture resistance (DTS) (46.31 MPa) than group 2 (37.14 MPa) and group 4 (40.91 MPa). ANOVA revealed a statistically significant difference (p =0.000) (Table 1).

**Table 1:** Intergroup comparison using one-way ANOVA test *Statistically significant ANOVA, analysis of variance

Groups	Number	Mean	Standard deviation	p-Value
Group 1	10	51.65 MPa	9.20 MPa	0.000*
Group 2	10	37.14 MPa	4.85 MPa	
Group 3	10	46.31 MPa	6.38 MPa	
Group 4	10	40.91 MPa	3.78 MPa	

The mean difference between groups 1 and 2 and groups 1 and 4 was highly significant (p = 0.000 and 0.001, respectively). The mean difference between groups 2 and 1 and groups 2 and 3 was highly significant (p = 0.000 and 0.003, respectively). The mean difference between groups 3 and 2 was highly significant (p = 0.003). The mean difference between groups 4 and 1 was highly significant (p = 0.001) (Table 2).

**Table 2:** Intragroup comparison assessed with post-hoc test *Statistically significant

Group A	Group B	Mean difference	p-Value
Group 1	Group 2	14.51 MPa	0.000*
	Group 3	5.33 MPa	0.070
	Group 4	10.74 MPa	0.001*
Group 2	Group 1	-14.51 MPa	0.000*
	Group 3	-9.17 MPa	0.003*
	Group 4	-3.77 MPa	0.195
Group 3	Group 1	-5.34 MPa	0.070
	Group 2	9.17 MPa	0.003*
	Group 4	5.40 MPa	0.067
Group 4	Group 1	-10.74 MPa	0.001*
	Group 2	3.77 MPa	0.195
	Group 3	-5.40 MPa	0.067

## Discussion

The design of the post and core specimens in this study represented a commonly encountered clinical setting with restricted inter-occlusal height. Clinically, when a core material is added to a post, it should reach approximately 2 mm above the post head. In some clinical scenarios, however, such extension is prohibited, and the post head must end flush with the top surface of the core [6,11]. Tests have shown that parallel-sided posts are more retentive than tapered posts, while threaded or serrated posts are more retentive than smooth posts [7,12]. As a result, this study used uniformly serrated and parallel posts to provide substantial retention for the composite core material [13,14]. The control group (group 1), which consisted of composite core building material with no posts, had the highest DTS values of all groups (51.65). Post incorporation in the core specimens resulted in a considerable drop in the specimens' DTS values, regardless of the type of post utilized. Using stainless steel, glass fiber, and carbon fiber posts in this research reduced the fracture resistance of the core build-up material by 28.1% for stainless steel posts, 10.4% for glass fiber posts, and 20.8% for carbon fiber posts. These findings show that the composite resin core material has superior fracture resistance when employed as a solid block. As a result, if the tooth has enough structural integrity, prefabricated posts should be questioned. As a result, this study's null hypothesis was not validated, as the composite resin core material without a post demonstrated higher DTS than the composite resin core material with a post. The current study's findings are consistent with those of Santos et al.’s [6]. When diametral tensile force was applied, the scientists discovered that using posts did not strengthen the composite resin core. They also confirmed that nonmetallic, fiber-reinforced posts had the greatest DTS values while stainless steel posts had the lowest. The current investigation also found that composite cores with two nonmetallic fiber posts had greater DTS values than stainless steel posts. The possibility is that glass fiber and carbon fiber posts were physically and chemically retained with the composite core via the serrated configuration of their heads and the application of a bonding agent.

On the other hand, the stainless steel post was just mechanically attached to the composite core. However, statistical analysis revealed that the mean difference in DTS values between carbon fiber posts (40.91 MPa) and stainless steel posts (37.14 MPa) was not statistically significant. One possible explanation is that adhesive application does not significantly improve the bond strength of core material to a carbon fiber post with a highly cross-linked epoxy resin matrix that lacks enough hydroxyl groups on their surface that can react with methacrylate groups of the composite resin core [5,15]. DTS values for composite core materials have also been reported by Cho et al. [16]. The specimens utilized in their investigation had the same diameter as those used in this study (6 mm). They got DTS values ranging from 51 to 55 MPa for two light-polymerized composite resin materials. The mean DTS of composite core (group 1) in the current investigation (51.65 MPa) is similar to the values reported in the previous study. The DTS of composite core (dual polymerized) material reported in this study was greater than the 35.9 MPa reported by Cohen et al. [17] for auto-polymerized composite resin core material (Ti-Core, Dental Essential Systems, South Hackensack, NJ) and the 44.6 MPa reported by Levartovsky et al. [18] for dual-polymerized composite resin core material (Fluorocore). Sadly, these materials are from an old generation of composites that have since been discontinued. According to Purton and Payne, tensile force tests were conducted on stainless steel serrated posts and smooth surface carbon fiber posts to determine the attachment of composite resin cores [13]. The stainless steel posts required more force to detach from the resin cores (65.6 kg) than the carbon fiber posts (38.9 kg). However, the strength of the tensile bond between cores and posts was highly dependent on the post configuration in that study. Carbon fiber posts had lower tensile values because the head of the posts lacked a retention feature, resulting in a weak bond between the composite core and the carbon fiber.

The present study has a limitation because the tests employed may not accurately reflect clinical conditions. They are harsher (more severe) than the forces experienced in the oral cavity. Tensile force is just one of numerous masticatory forces in the oral cavity. Situations in which pure tensile stresses are used are uncommon. Furthermore, the post and core are protected by a crown, which helps distribute masticatory pressure more uniformly across the root, post, and core complex. Such a study provides us with the relative order of the tested properties. However, additional research is required to correlate this study's implications to clinical success precisely.

## Conclusions

Prefabricated posts bonded to resin composite core material, such as stainless steel, carbon fiber, and glass fiber, decreased resin composite core material fracture resistance by 28.1%, 20.8%, and 10.4%, respectively. A composite core with stainless steel post systems displayed significantly lower mean DTS values. In contrast, glass fiber and carbon fiber post systems significantly increased DTS values. Based on the limitations of this study, it could be concluded that glass fiber reinforced prefabricated posts are an appropriate material to use when the composite resin is used as a core build-up.
